# Immune alveolitis in interstitial lung disease: an attractive cytological profile in immunocompromised patients

**DOI:** 10.1186/s12890-022-01871-w

**Published:** 2022-03-05

**Authors:** Antoine Moui, Stéphanie Dirou, Christine Sagan, Renan Liberge, Claire Defrance, Pierre-Paul Arrigoni, Olivier Morla, Christine Kandel-Aznar, Laurent Cellerin, Arnaud Cavailles, Emmanuel Eschapasse, Florent Morio, Pierre-Antoine Gourraud, Thomas Goronflot, Adrien Tissot, François-Xavier Blanc

**Affiliations:** 1 Service de Pneumologie, l’Institut du Thorax, Hôpital Guillaume et René Laënnec, Boulevard Jacques Monod, Centre Hospitalier Universitaire de Nantes, 44093 Nantes, France; 2 Service d’Anatomopathologie, Hôpital Hôtel-Dieu, Centre Hospitalier Universitaire de Nantes, Nantes, France; 3 Service d’Imagerie Médicale, Hôpital Hôtel-Dieu, Centre Hospitalier Universitaire de Nantes, Nantes, France; 4 Laboratoire de Parasitologie-Mycologie, Institut de Biologie, Centre Hospitalier Universitaire de Nantes, Nantes, France; 5 Pôle Hospitalo-Universitaire 11: Santé Publique, Clinique des données, Inserm CIC 1413, Centre Hospitalier Universitaire de Nantes, Nantes, France

**Keywords:** Bronchoalveolar lavage, Immune alveolitis, Interstitial lung disease, Immunosuppression, *Pneumocystis* pneumonia

## Abstract

**Background:**

Bronchoalveolar lavage (BAL) is a major diagnostic tool in interstitial lung disease (ILD). Its use remains largely quantitative, usually focused on cell differential ratio. However, cellular morphological features provide additional valuable information. The significance of the “immune alveolitis” cytological profile, characterized by lymphocytic alveolitis with activated lymphocytes and macrophages in epithelioid transformation or foamy macrophages desquamating in cohesive clusters with lymphocytes, remains unknown in ILD. Our objective was to describe patients’ characteristics and diagnoses associated with an immune alveolitis profile in undiagnosed ILD.

**Methods:**

We performed a monocentric retrospective observational study. Eligible patients were adults undergoing diagnostic exploration for ILD and whose BAL fluid displayed an immune alveolitis profile. For each patient, we collected clinical, radiological and biological findings as well as the final etiology of ILD.

**Results:**

Between January 2012 and December 2018, 249 patients were included. Mean age was 57 ± 16 years, 140 patients (56%) were men, and 65% of patients were immunocompromised. The main etiological diagnosis was *Pneumocystis* pneumonia (PCP) (24%), followed by drug-induced lung disease (DILD) (20%), viral pneumonia (14%) and hypersensitivity pneumonitis (HP) (10%). All PCP were diagnosed in immunocompromised patients while HP was found in only 8% of this subgroup. DILD and viral pneumonia were also commonly diagnosed in immunocompromised patients (94% and 80%, respectively).

**Conclusion:**

Our study highlights the additional value of BAL qualitative description in ILD. We suggest incorporating the immune alveolitis profile for the diagnosis and management of ILD, especially in immunocompromised patients, since it guides towards specific diagnoses.

**Supplementary Information:**

The online version contains supplementary material available at 10.1186/s12890-022-01871-w.

## Background

Interstitial lung diseases (ILD) have heterogeneous etiologies. Usually, they are separated between ILD of known causes and idiopathic ILD. A large epidemiological study emphasized the importance of secondary ILDs relative to idiopathic ILD that have been classified by the American Thoracic Society (ATS) and the European Respiratory Society (ERS) [1–3]. The etiological diagnostic approach of ILD can be difficult and requires a rigorous clinical examination, serological tests and a high resolution lung CT scan with thin section (< 2 mm) [4–8]. A likely diagnosis can be suggested by specific CT scan patterns [9]. In addition to CT scan analysis, a bronchoalveolar lavage (BAL) may be of great help to rule out differential diagnosis, in particular infectious diseases.

According to the nature of increased BAL fluid cell type (or alveolitis), different quantitative cytological profiles have been identified: lymphocytic (> 15% lymphocytes), neutrophilic (> 3% neutrophils) and eosinophilic alveolitis (> 1% eosinophils). However, none is specific of a single type of ILD [10]. Typical BAL findings allow to obtain a formal diagnosis in some rare ILD such as pulmonary alveolar proteinosis, lipoid pneumonia and acute eosinophilic pneumonia [11]. When analyzed together with clinical, biological and radiological data, examination of BAL has an added diagnostic value and guides towards a selection of disease. For example, lymphocytic alveolitis can be found in hypersensitivity pneumonitis (HP) or sarcoidosis [12, 13], whereas neutrophilic alveolitis rather suggests idiopathic pulmonary fibrosis or asbestosis [10]. However, apart from macrophages with smoking related inclusions or foamy macrophages, qualitative morphological analysis of BAL cells remains poorly described.

Immune alveolitis is a morphological profile of BAL fluid characterized by an abundant cellularity, with high lymphocytes rates between 30 and 80% (rather CD8+, activated lymphocytes with more abundant cytoplasm), some eosinophilic and neutrophilic polynuclear cells, particular mast cells and macrophages that can be described as ‘foamy’ and/or ‘in epithelioid transformation’, desquamating into cohesive clusters [14]. There is minimal literature describing such profile in HP, reflecting the pulmonary immune reaction that occurs after allergen inhalation in sensitized individuals [15–17].

We hypothesized that immune alveolitis could also be taken into account in the diagnostic approach of ILD and restrict the suspected etiologies. We conducted the present study to evaluate the etiologies’ frequency of ILD in patients with such immune alveolitis profile on BAL. Our secondary objective was to assess the association of clinical, radiological and biological factors to ILD final etiological diagnosis in this population.

## Methods

### Study design and patients

In this observational, descriptive, retrospective and monocentric study conducted at the University Hospital of Nantes, France, from January 2012 to December 2018, all adults who presented with ILD, detected on a chest radiograph or a CT scan, and who underwent a BAL that revealed an immune alveolitis profile were selected by automated file extraction of medical records. BAL was performed using 90 mL of saline delivered into a lung segment affected by interstitial disease as identified by CT scan. Then, fluid was retrieved using an adjusted negative suction pressure with a target of a minimal volume ≥ 30%. BAL sample was separated in at least 4 aliquots to be sent for microbiological analyzes and for cytological examination. BAL fluid was centrifuged. Differential cell counts were obtained from slide stained with May-Grünwald-Giemsa. At least 200 cells were counted in each subject. The number of ciliated or squamous epithelial cells was noted but not included in the differential count. An immune alveolitis profile on BAL was defined by the combination of lymphocytosis (greater than 10%) and the following morphological criteria: activated lymphocytes, epithelioid transformation of macrophages, desquamation of macrophages into cohesive clusters, foamy macrophages (intra-cytoplasmic vacuoles).

For this study, patients were considered as being immunocompromised if they had a solid organ or bone marrow transplantation, or received treatment for solid or hematological cancer, or were treated by corticosteroids (at a daily dose ≥ 20 mg for at least three weeks) or any other immunosuppressive drugs.

### Endpoints

The primary endpoint was the etiologies’ frequency of ILD with an immune alveolitis profile on BAL. The final etiological diagnosis was collected in the electronic medical record until July 2019. Indeed, some etiologies were established several months after initial investigations. Some of these diagnoses may have been retained after multidisciplinary discussion or after a lung biopsy. For the uncertain diagnoses, all medical records were reviewed by an adjudication committee composed of a pulmonologist and a senior pathologist. The diagnosis of viral pneumonia was retained even in the absence of microbiological documentation when the clinical context, paraclinical data and clinical evolution were consistent with this diagnosis. In patients with intermediate fungal loads, *Pneumocystis* pneumonia (PCP) diagnosis was retained when the serum β-D glucans were either positive or after multidisciplinary discussion when unavailable. As secondary endpoints, we analyzed clinical, radiological and biological characteristics of patients and assessed etiologies’ frequency in particular subpopulations.

### Ethics

The study protocol was submitted and approved by the «Délégation à la recherche clinique et à l’innovation (DRCI)» of our institution and by the «Institutional Review Board of the French-speaking Respiratory Medicine Society (Société de Pneumologie de Langue Française, SPLF)».

### Statistical analysis

All analyses were conducted on the R software (version 3.3.0). Continuous variables were described according to their mean and standard deviation. Categorical variables were described as number and percentage. A univariate descriptive analysis was carried out to describe the overall population and to identify variables associated with the final etiological diagnosis (after excluding uncertain or infrequent diagnoses). We performed univariate analyses for each data item with the 5 most common etiologies. “Uncertain diagnoses” were excluded to minimize bias in the search for predictive factors. “Other diagnoses” and mycobacteria were excluded because of a small number of patients. We used the Chi-square independence test to assess the significance of the association between two categorical variables when validity test conditions were met. Otherwise, Fisher exact test was used. For continuous variables, the homogeneity of the variances and the normal distribution of the variables were first tested by the Levene and Shapiro–Wilk tests, respectively. Significance of differences in means was studied using Student’s *t*-test when two means were compared or using a one-factor ANOVA when more than two means were compared. If conditions for applying these tests were not respected (normal distribution), we respectively used the Mann–Whitney–Wilcoxon test and the non-parametric Kruskal–Wallis test. We considered the statistical significance threshold for all tests at 5%. Multiple testing issue was tackled using the Benjamini–Hochberg method by limiting false discovery rate to 5%.

## Results

### Patients

During the study period, 274 patients presented with immune alveolitis, as diagnosed by the pathologist. Among them, 25 were excluded because they did not meet the study criteria: 11 without ILD and 14 who were managed in another center and were only referred for bronchoscopy. Thus, 249 patients were analyzed.

Mean age of patients was 57 ± 16 years old and 140 (56%) were men (Table 1). Ninety-eight patients (40%) were current or former smokers. A total of 163 patients (65%) received treatment for solid or hematological cancer, had transplantation or immunosuppressive therapy and were therefore considered as being immunocompromised. Corticosteroid was the most common immunosuppressive therapy (30% of patients) with an average daily dose of 16.5 mg (prednisone equivalent). PCP prophylaxis was given in 43 patients (17% of the general population and 26% of immunocompromised patients) and cotrimoxazole was the most frequently used drug (24 patients).

**Table 1:** Clinical, biological and radiological characteristics of patients

Characteristics of patients	Total (N = 249)
Clinical	
Age, years	57 ± 16
Male	140 (56)
Smoking status	
Smoker (*NA* = *3*)	98 (40)
Number of pack-years	21 ± 18
Comorbidities	
Immunocompromised	163 (65)
Solid cancer	65 (26)
Hematological cancer	56 (22)
Solid organ transplant	39 (16)
Bone marrow transplant	24 (10)
Connective tissue diseases	20 (8)
HIV positive	8 (3)
Treatments	
Corticosteroid	75 (30)
Dose, mg/day	16.5 ± 16
Methotrexate	18 (7)
Mycophenolate mofetil	20 (8)
Ciclosporin	20 (8)
Chemotherapy	29 (12)
Immunotherapy	11 (4)
*Pneumocystis* prophylaxis	43 (17)
Radiological	
Lesions on chest CT scan (*NA* = *21*)	
Ground glass opacities	179 (79)
Reticulation	88 (39)
Micronodules	66 (29)
Consolidation	62 (27)
Septa thickening	37 (16)
Mosaic attenuation	11 (5)
Bilateral lesions	197 (86)
Distribution (*NA* = *21*)	
Diffuse	136 (60)
Lower lobes	54 (24)
Upper lobes	32 (14)
Biological	
Serum biology	
Leukocytes, giga/L (*NA* = *36*)	8.0 ± 6.0
Neutrophils, giga/L (*NA* = *47*)	5.5 ± 4.0
Lymphocytes, giga/L (*NA* = *47*)	1.6 ± 3.7
Eosinophils, giga/L (*NA* = *48*)	0.18 ± 0.21
Hemoglobin, g/dL (*NA* = *36*)	12.1 ± 2.2
Platelets, giga/L (*NA* = *39*)	278 ± 561
CRP, mg/dL (*NA* = *92*)	65.4 ± 73.2
Bronchial fibroscopy	
Bacteria	37 (15)
Mycobacteria (*NA* = *4*)	7 (3)
Positive viral PCR (*NA* = *18*)	34 (15)
Fungi	94 (38)
*Pneumocystis* cysts (direct examination)	17 (7)
Positive *Pneumocystis* PCR (*NA* = *75*)	89 (51)
*Pneumocystis* PCR copies (*NA* = *4*)	
Colonization	32 (38)
Intermediate	25 (29)
Infection	28 (33)
BAL cellularity, cells/ml	245,692 ± 350,317
Cell populations on BAL, % (*NA* = *4*)	
Macrophages	43 ± 17
Lymphocytes	51 ± 18
Neutrophils	5 ± 8
Eosinophils	1.5 ± 4
Morphological abnormalities on BAL, (*NA* = *1*)	
Activated lymphocytes	238 (96)
Macrophages into cohesive clusters	245 (99)
Epithelioid transformation of macrophages	240 (97)
Foamy macrophages	185 (75)

Data are presented as mean ± SD or N (%)

*BAL* bronchoalveolar lavage, *SD* standard deviation, *N* number, *NA* not applicable

### Clinical, radiological, biological and BAL features

The most frequent clinical signs were dyspnea (75%), cough (58%) and fever (38%). Extra-thoracic signs (skin, eye, joint, muscle) were not uncommon (15%).

Radiological patterns were heterogeneous in these patients with immune alveolitis (Table 1). Ground glass opacities were the most frequently observed (79%), preferentially bilateral (86%) and diffuse (60%). In addition, the 21 patients whose CT scan was not performed all exhibited an interstitial syndrome on chest radiography.

Blood cell counts were normal in most patients, notably with the absence of eosinophilia and with a lymphocyte rate within the lower limits of normal (Table 1). *Pneumocystis jirovecii* PCR was positive in half of the tested population (n = 89 patients).

**Fig. 1:**
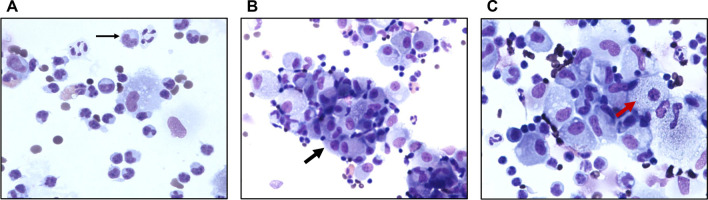
Typical morphological characteristics of immune alveolitis on bronchoalveolar lavage. Activated lymphocytes (thin black arrow) (**A**), desquamation of macrophages into cohesive clusters (thick black arrow) (**B**), epithelioid transformation of macrophages and foamy macrophages (intra-cytoplasmic vacuoles) (red arrow) (**C**). May-Grünwald Giemsa ×40

Cytological analysis of BAL fluid found a high cellularity of 245,692 ± 350,317 cells/mL. Quantitative analysis of BAL cell populations revealed a lymphocytosis (51 ± 18%), a rate of macrophages reduced to 43 ± 17% and a rate of neutrophils and eosinophils slightly higher than normal (5 ± 8% and 1 ± 4%, respectively). Morphological analysis almost always showed activated lymphocytes (96%), desquamative macrophages into cohesive clusters (99%) and macrophages in epithelioid transformation (97%) (Fig. 1). Presence of foamy or micro-vacuolated macrophages was frequent (75%). Quantitative and qualitative BAL analyses were not different according to smoking status (Additional file 1).

### Primary outcome

**Fig. 2:**
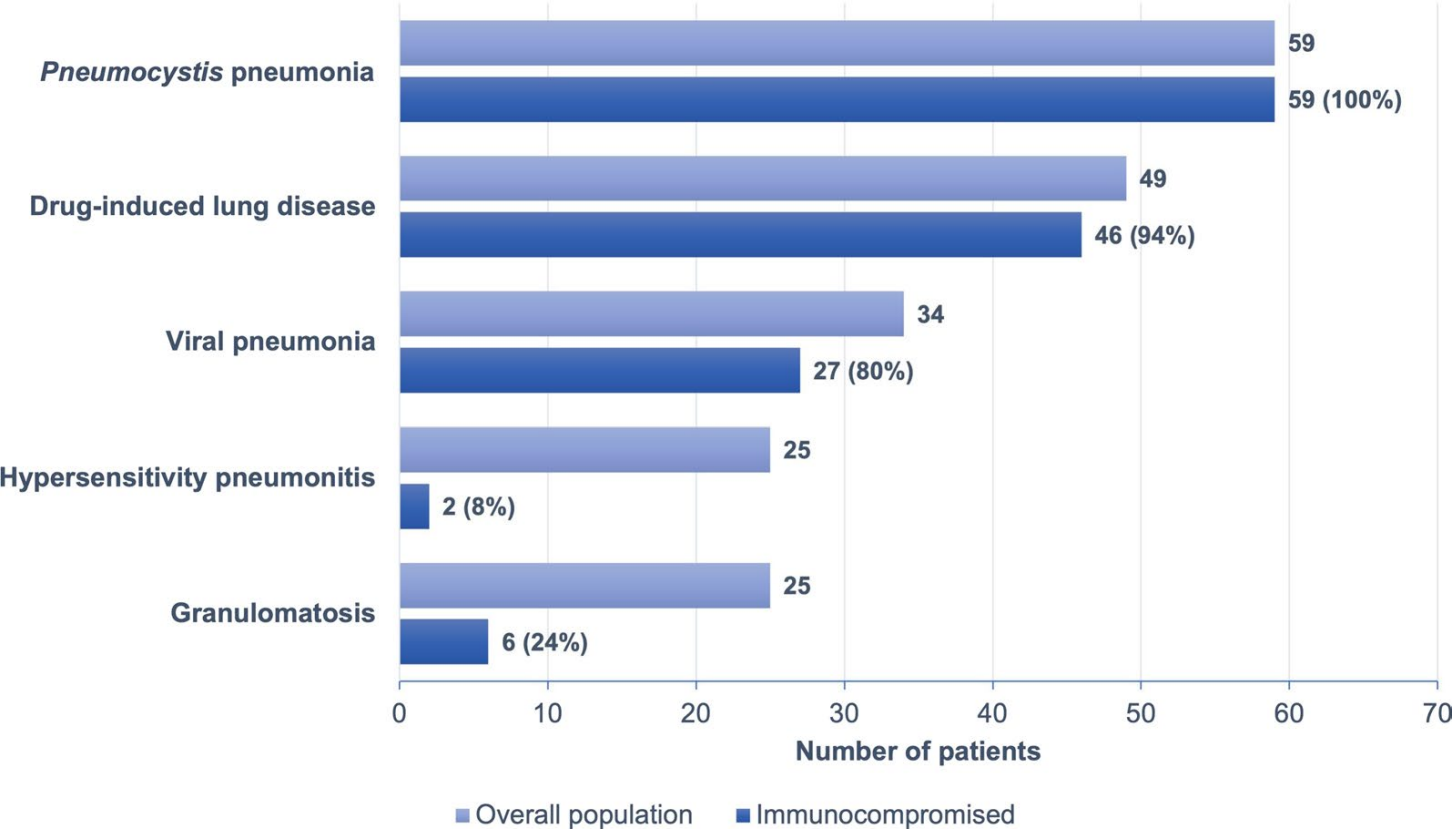
Distribution of etiological diagnoses in the overall population and immunocompromised patients

Etiological diagnoses of ILD associated with an immune alveolitis profile are shown in Fig. 2 and Table 2. The most common diagnosis was *Pneumocystis* pneumonia in 59 patients (24%), followed by drug-induced lung disease (DILD) in 49 patients (20%), everolimus being the most frequently involved drug (n = 11 patients), followed by nivolumab (n = 5) and methotrexate (n = 5). Amiodarone was associated with DILD in only 3 patients (6%). Thirty-four patients (14% of the global population) had viral pneumonia with viral identification in half of cases, respiratory syncitial virus, coronavirus and rhinovirus being the most frequently identified viruses (Additional file 2).

**Table 2:** Etiological diagnoses in the overall population

Etiological diagnosis	Total (N = 249)
*Pneumocystis* pneumonia	59 (24)
Drug induced lung disease	49 (20)
Viral pneumonia	34 (14)
Uncertain diagnoses	26 (10)
Hypersensitivity pneumonitis	25 (10)
Granulomatosis	25 (10)
Sarcoidosis	19 (8)
Common variable immunodeficiency	2 (1)
Other granulomatosis	4 (1)
Other diagnoses	17 (7)
Connective tissue disease	5 (2)
Vasculitis	3 (1)
Pulmonary graft versus host disease	3 (1)
Bacteria (intracellular)	2 (1)
Idiopathic nonspecific interstitial pneumonia	1 (0.5)
Cryptogenic organizing pneumonia	1 (0.5)
Lymphoma	1 (0.5)
Silicosis	1 (0.5)
Mycobacteria	14 (6)
*Mycobacterium tuberculosis*	11 (5)
Non-tuberculous mycobacterium	3 (1)

Data are presented as N (%)

*SD* standard deviation, *N* number

Ten percent of patients were diagnosed with HP (n = 25), granulomatosis (n = 25), or had uncertain diagnosis despite assessment by the adjudication committee (n = 26) (Table 2).

### Immunocompromised subpopulations

Among the 163 immunocompromised patients, the main diagnosis was *Pneumocystis* pneumonia (n = 59 patients, 36%). All patients with a final diagnosis of PCP were immunocompromised compared to 8% of patients with HP.

Twenty-six percent of the immunocompromised patients received PCP prophylaxis and 13 patients were diagnosed with PCP despite such prophylaxis. Four of them were taking cotrimoxazole with uncertain compliance while the 9 others received nebulized pentamidine or atovaquone. In immunocompromised patients, the second most common diagnosis was DILD (46 patients, 28%). Viral pneumonia was diagnosed in 27 patients (16%). The distribution of diagnoses was similar for patients receiving corticosteroids (75 patients, 30% of the global population). Among them, 31% had a daily dose > 15 mg equivalent prednisone and 26 patients received PCP prophylaxis (35%). No patient taking corticosteroids was diagnosed with HP.

### Associated factors with the etiological diagnosis

Clinical, radiological and biological factors associated with the final etiological diagnosis are shown in Table 3, Additional file 3 and Additional file 4. In univariate analysis, age, solid cancer, hematological cancer, solid organ or bone marrow transplantation, corticosteroid therapy, chemotherapy, or immunotherapy, immunosuppression, PCP prophylaxis, the presence of fever, dyspnea or extra-thoracic signs were all associated with immune alveolitis (Table 3). Radiological factors associated with the etiological diagnosis were the presence of ground glass opacities, micronodules, condensations or a mosaic attenuation (Additional file 3). Finally, biological factors associated with this profile were white and red blood cells count, and the positivity of microbiological examinations (Additional file 4).

**Table 3:** Clinical characteristics of patients according to etiology

Clinical characteristics (N = 192)	PCP (N = 59)	DILD (N = 49)	Viral pneumonia (N = 34)	HP (N = 25)	Granuloma-tosis (N = 25)	P*
Age (years)	57 ± 16	65 ± 13	54 ± 17	60 ± 14	50 ± 15	**0.001**
Male	29 (49)	26 (53)	17 (50)	16 (64)	20 (80)	0.08
Smoking status						
Smoker (*NA* = *3*)	24 (40)	21 (43)	10 (29)	8 (32)	12 (48)	0.5
Pack-years	18 ± 14	30 ± 30	23 ± 18	14 ± 16	21 ± 16	0.5
Comorbidities						
Immunocompromised	59 (100)	46 (94)	27 (80)	2 (8)	6 (24)	**0.0001**
Solid cancer	18 (32)	27 (56)	7 (21)	5 (20)	1 (4)	**0.0005**
Hematological cancer	22 (37)	7 (14)	15 (45)	0 (0)	0 (0)	**0.0001**
Solid organ transplant	19 (31)	7 (15)	7 (21)	0 (0)	1 (4)	**0.0008**
Bone marrow transplant	9 (15)	0 (0)	8 (23)	0 (0)	0 (0)	**0.0004**
Connective tissue disease	6 (11)	6 (13)	1 (3)	1 (4)	0 (0)	0.3
HIV	4 (7)	1 (2)	0 (0)	0 (0)	0 (0)	0.3
Treatments						
Corticosteroids	31 (52)	17 (34)	12 (35)	0 (0)	5 (20)	**0.0005**
Dose, mg/day	20 ± 19	13 ± 10	12 ± 9	–	11 ± 3	0.7
Methotrexate	6 (10)	6 (12)	2 (6)	0 (0)	2 (8)	0.4
Mycophenolate mofetil	9 (15)	2 (4)	4 (12)	0 (0)	0 (0)	0.03
Ciclosporin	6 (10)	4 (8)	8 (23)	0 (0)	1 (4)	0.04
Chemotherapy	12 (20)	14 (28)	2 (5)	0 (0)	0 (0)	**0.0002**
Immunotherapy	3 (5)	7 (14)	0 (0)	0 (0)	0 (0)	**0.02**
Pneumocystis prophylaxis	13 (21)	3 (6)	15 (44)	0 (0)	1 (4)	**0.0001**
Physical examination						
Fever	39 (66)	15 (30)	27 (79)	2 (8)	2 (8)	**0.0001**
Deterioration of general condition	9 (15)	5 (10)	6 (17)	4 (16)	3 (12)	0.9
Cough	31 (52)	28 (57)	21 (62)	18 (72)	11 (44)	0.3
Dyspnea	48 (81)	44 (89)	28 (82)	21 (84)	14 (56)	**0.02**
Expectorations	12 (20)	7 (14)	9 (26)	7 (28)	4 (16)	0.5
Extra-thoracic signs	2 (3)	4 (8)	2 (6)	4 (16)	14 (56)	**0.0001**

Bold was used when the statistically significant threshold was reached

Data are presented as mean ± SD or N (%)

*SD* standard deviation, *N* number, *NA* not applicable, *PCP* *Pneumocystis* pneumonia, *DILD* Drug-induced lung disease, *HP* Hypersensitivity pneumonitis

*Multiple testing issue was tackled using Benjamini–Hochberg method by limiting False Discovery Rate to 5%. Statistical significance threshold was at 3%

## Discussion

In this retrospective analysis of 249 patients with an immune alveolitis profile on BAL, the main five ILD’s etiologies were *Pneumocystis* pneumonia (24%), followed by DILD (20%), viral pneumonia (14%), HP (10%) and granulomatosis (10%). Immunocompromised patients represented 65% of the overall population. In this subgroup, the most frequent diagnosis was by far PCP and HP diagnosis was retained in only two cases.

To the best of our knowledge, the diagnostic contribution of the immune alveolitis morphological profile has not been previously described in patients with PCP. However, lymphocytic alveolitis is commonly reported in PCP and prognosis relating to BAL cellular analysis has been evaluated [18]. Lymphocytosis was found with an average rate of 31% in 166 non-HIV infected patients with PCP [19], which is consistent with our results. In addition, BAL cell type profile seems to have a prognostic value. In non-HIV infected patients, Lee et al*.* evaluated the prognosis impact of BAL cell profile in PCP. Alveolar lymphocytes appeared to be lower in patients with severe PCP compared to those with mild and moderate disease [19]. Recently, Gaborit et al*.* analyzed prognostic factors in immunocompromised patients with *Pneumocystis* pneumonia [20]. The presence of an immune alveolitis profile on BAL was an independent protective factor for mortality at 90 days. Based on these observations, additional investigations to evaluate the prognostic contribution of this profile in other ILDs are warranted.

Immune alveolitis profile on BAL has been yet poorly explored. It is usually considered as an immuno-allergic profile, which refers to the pathophysiology that was mainly described in HP during the 90’s [15–17]. Recent ATS/ERS guidelines focus on lymphocyte counts and recommend to obtain BAL fluid in cases with suggestive diagnosis of HP [21]. Even though a 40% lymphocyte threshold has been identified as an important item for the diagnosis of HP [22], ATS/ERS guidelines do not set a lymphocyte threshold. Furthermore, immune alveolitis profile has not been detailed but could provide an additional value in distinguishing HP from others ILD related entities.

Many heterogeneous BAL cytological features can be associated with pulmonary drug toxicity and hamper BAL contribution in the diagnostic approach of DILD [23], Morphological description of BAL cells had focused on intra alveolar foamy macrophages in amiodarone pneumonitis [24]. Apart from amiodarone (implicated in only 6% of DILD in our series), drugs that were the most frequently involved in our study were everolimus, followed by nivolumab and methotrexate.

The cytological profile of BAL has been well described in sarcoidosis and is characterized by a rather moderate lymphocytic alveolitis (about 30%) that may reach higher levels (50%) when the disease is active [25]. The significant proportion of granulomatosis associated with an immune alveolitis profile, and especially sarcoidosis, is an unexpected result of our study. In some patients with a past history of sarcoidosis, immune alveolitis was found in a context of disease recurrence, leading to a resumption of immunosuppressive therapies. In view of these findings, immune alveolitis would be more likely present in the early and active phases of the disease.

Ten percent of the population did not have a definite etiological diagnosis at the end of data collection, which highlights the difficulty associated to the ILD diagnostic work-up. BAL is a recognized diagnostic tool to investigate ILD [10]. When BAL is interpreted in combination with clinical data and HRCT findings, it holds a great potential in establishing ILD’s etiology. Validation of a new BAL morphological pattern will hopefully aim to reduce ILD differential diagnoses and limit the need for surgical lung biopsy. Indeed, the 5 most common diagnoses accounted for nearly 80% of the final etiologies in our study. In addition, when the immunocompromised status was considered, the main final etiologies were reduced to three: PCP, DILD and viral pneumonia.

Results from univariate analysis highlight clinical, radiological or biological factors that can help in the diagnostic process and consequently that need to be sought. For example, fever or the absence of extra thoracic signs seem indicative factors to reduce ILD etiologies, while corticosteroids use appears to be negatively associated with HP.

Our study had some limitations. Its retrospective design led to missing data, especially regarding the search for different antibodies (e.g., anti-nuclear, or serum precipitins). Another study limitation was a potential selection bias related to its monocentricity. Indeed, our tertiary hospital is a reference center for kidney, heart, lung and bone marrow transplants. As a consequence, 65% of our population was immunocompromised. This parameter had obviously an impact on the frequency of final etiologies, especially for PCP.

## Conclusion

In summary, this study highlights the additional value of BAL qualitative description in ILD with a detailed characterization of immune alveolitis, a poorly studied BAL profile. We suggest incorporating this profile for the diagnosis and management of ILD, especially in immunocompromised patients. Indeed, the presence of an immune alveolitis profile reduces the etiological possibilities and should systematically lead to exclude the diagnosis of PCP. The diagnostic contribution of immune alveolitis is inseparable of clinical, radiological and biological data that must be taken into account in a multidisciplinary diagnostic process.

## Supplementary Information

**Additional file 1.** Characteristics of bronchoalveolar lavage according to smoking status. Data are presented as mean ± SD or N (%). *Smoking status is missing for 3 patients. Definition of abbreviations: BAL = bronchoalveolar lavage; N = number; NA = not applicable.

**Additional file 2.** Viruses’ identification of viral pneumonia. Data are presented as N (%). N number.

**Additional file 3.** Radiological characteristics of patients according to etiology and univariate analysis. Data are presented as mean ± SD or N (%). *Multiple testing issue was tackled using Benjamini–Hochberg method by limiting False Discovery Rate to 5%. Statistical significance threshold was at 3%. DILD Drug-induced lung disease; HP Hypersensitivity pneumonitis; N number; NA not applicable; PCP *Pneumocystis* pneumonia.

**Additional file 4.** Biological characteristics of patients according to etiology. Data are presented as mean ± SD or N (%). *Multiple testing issue was tackled using Benjamini–Hochberg method by limiting False Discovery Rate to 5%. Statistical significance threshold was at 3%. DILD Drug-induced lung disease; HP Hypersensitivity pneumonitis; N number; NA not applicable; PCP *Pneumocystis* pneumonia.

## Data Availability

The datasets used and/or analyzed during the current study are available from the corresponding author on reasonable request.

## Abbreviations

ATS: American Thoracic Society
BAL: Bronchoalveolar lavage
DILD: Drug-induced lung disease
ERS: European Respiratory Society
HP: Hypersensitivity pneumonitis
ILD: Interstitial lung disease
PCP: *Pneumocystis* Pneumonia
