# Supplementary material for: Immune alveolitis in interstitial lung disease: an attractive cytological profile in immunocompromised patients
Source: BMC Pulm Med. 2022 Mar 5;22:79. doi: 10.1186/s12890-022-01871-w (PMC8897721; doi:10.1186/s12890-022-01871-w)
Supplement: Supplementary file 1 — Additional file 1. Characteristics of bronchoalveolar lavage according to smoking status. Data are presented as mean ± SD or N (%). *Smoking status is missing for 3 patients. Definition of abbreviations: BAL = bronchoalveolar lavage; N = number; NA = not applicable. [file 12890_2022_1871_MOESM1_ESM.docx]

**Additional File 1. Characteristics of bronchoalveolar lavage according to smoking status.**

| BAL characteristics | Current / former  smokers (N=98) | Non-smokers *  (N=148) | *P* |
| --- | --- | --- | --- |
| BAL cellularity, cells/mL | 210,484 ± 264,107 | 263,295 ± 386,966 | 0.55 |
| Cell populations, % (*NA=4)* |  |  |  |
| Macrophages | 42 ± 17 | 43 ± 17 | 0.73 |
| Lymphocytes | 50 ± 19 | 51 ± 18 | 0.96 |
| Neutrophils | 5 ± 8 | 5 ± 8 | 0.72 |
| Eosinophils | 2 ± 5 | 1 ± 2 | 0.97 |
| Morphological abnormalities, *(NA=1)* |  |  |  |
| Activated lymphocytes | 92 (94) | 143 (96) | 0.35 |
| Macrophages into cohesive clusters | 95 (97) | 147 (99) | 0.30 |
| Epithelioid transformation of macrophages | 94 (96) | 144 (97) | 0.72 |
| Foamy macrophages | 74 (75) | 109 (74) | 0.76 |

Data are presented as mean ± SD or N (%). * Smoking status is missing for 3 patients. Definition of abbreviations: BAL = bronchoalveolar lavage; N = number; NA = not applicable.
