# Supplementary material for: Immune alveolitis in interstitial lung disease: an attractive cytological profile in immunocompromised patients
Source: BMC Pulm Med. 2022 Mar 5;22:79. doi: 10.1186/s12890-022-01871-w (PMC8897721; doi:10.1186/s12890-022-01871-w)
Supplement: Supplementary file 2 — Additional file 2. Viruses’ identification of viral pneumonia. Data are presented as N (%). N number. [file 12890_2022_1871_MOESM2_ESM.docx]

**Additional File 2. Viruses’ identification of viral pneumonia.**

| Virus | Total (N=17) |
| --- | --- |
| Respiratory syncitial virus | 3 (18) |
| Coronavirus and Rhinovirus | 3 (18) |
| Coronavirus | 2 (12) |
| Cytomegalovirus | 2 (12) |
| Metapneumovirus | 2 (12) |
| Rhinovirus | 1 (6) |
| Influenza virus | 1 (6) |
| Parainfluenza virus | 1 (6) |
| Epstein-Barr virus | 1 (6) |
| Adenovirus | 1 (6) |

Data are presented as N (%). Abbreviations: N number
