# Supplementary material for: Immune alveolitis in interstitial lung disease: an attractive cytological profile in immunocompromised patients
Source: BMC Pulm Med. 2022 Mar 5;22:79. doi: 10.1186/s12890-022-01871-w (PMC8897721; doi:10.1186/s12890-022-01871-w)
Supplement: Supplementary file 3 — Additional file 3. Radiological characteristics of patients according to etiology and univariate analysis. Data are presented as mean ± SD or N (%). *Multiple testing issue was tackled using Benjamini–Hochberg method by limiting False Discovery Rate to 5%. Statistical significance threshold was at 3%. DILD Drug-induced lung disease; HP Hypersensitivity pneumonitis; N number; NA not applicable; PCP Pneumocystis pneumonia. [file 12890_2022_1871_MOESM3_ESM.docx]

**Additional File 3. Radiological characteristics of patients according to etiology and univariate analysis.**

| Radiological characteristics (N= 192) | PCP  (N=59) | DILD  (N=49) | Viral pneumonia  (N=34) | HP  (N=25) | Granuloma-tosis  (N=25) | *P ** |
| --- | --- | --- | --- | --- | --- | --- |
| Chest CT lesions, N (%) *(NA=20)* |  |  |  |  |  |  |
| Ground glass opacities | 49 (96) | 25 (81) | 27 (90) | 24 (96) | 11 (47) | **0.0005** |
| Micronodules | 7 (14) | 2 (5) | 9 (30) | 8 (32) | 17 (74) | **0.0005** |
| Reticulations | 14 (27) | 19 (44) | 9 (30) | 15 (60) | 9 (39) | 0.06 |
| Septa thickening | 8 (16) | 9 (21) | 4 (13) | 4 (16) | 3 (13) | 0.9 |
| Condensations | 8 (16) | 15 (34) | 12 (40) | 1 (4) | 4 (17) | **0.004** |
| Mosaic attenuation | 0 (0) | 1 (2) | 1 (3) | 5 (20) | 2 (8) | **0.004** |
| Bilateral lesion, N (%) | 49 (83) | 39 (79) | 27 (79) | 23 (92) | 20 (80) | 0.6 |
| Distribution, N (%) *(NA=21)* |  |  |  |  |  |  |
| Diffuse | 40 (68) | 25 (51) | 19 (55) | 14 (56) | 15 (60) | 0.5 |
| Lower lobes | 6 (11) | 10 (20) | 8 (23) | 8 (32) | 5 (20) | 0.1 |
| Upper lobes | 5 (8) | 8 (16) | 2 (9) | 3 (12) | 3 (12) | 0.6 |

Data are presented as mean ± SD or N (%). ***** Multiple testing issue was tackled using Benjamini-Hochberg method by limiting False Discovery Rate to 5%. Statistical significance threshold was at 3%.

Abbreviations: DILD Drug-induced lung disease; HP Hypersensitivity pneumonitis; N number; NA not applicable; PCP *Pneumocystis* pneumonia
