# Supplementary material for: Immune alveolitis in interstitial lung disease: an attractive cytological profile in immunocompromised patients
Source: BMC Pulm Med. 2022 Mar 5;22:79. doi: 10.1186/s12890-022-01871-w (PMC8897721; doi:10.1186/s12890-022-01871-w)
Supplement: Supplementary file 4 — Additional file 4. Biological characteristics of patients according to etiology. Data are presented as mean ± SD or N (%). *Multiple testing issue was tackled using Benjamini–Hochberg method by limiting False Discovery Rate to 5%. Statistical significance threshold was at 3%. DILD Drug-induced lung disease; HP Hypersensitivity pneumonitis; N number; NA not applicable; PCP Pneumocystis pneumonia. [file 12890_2022_1871_MOESM4_ESM.docx]

**Additional File 4. Biological characteristics of patients according to etiology.**

| Biological characteristics (N=192) | PCP  (N=59) | DILD  (N=49) | Viral pneumonia  (N=34) | HP  (N=25) | Granuloma-tosis  (N=25) | *P ** |
| --- | --- | --- | --- | --- | --- | --- |
| Blood counts |  |  |  |  |  |  |
| Leukocytes, giga/L *(NA= 36)* | 8.7 ± 7.8 | 7.7 ± 3.4 | 7± 4.2 | 9.3 ± 3.1 | 5.5 ± 2.3 | **0.008** |
| Neutrophiles, giga/L *(NA= 47)* | 5.9 ± 4.1 | 5.6 ± 3.3 | 4.6 ± 3.2 | 6.1 ± 2.8 | 3.5 ± 1.7 | **0.02** |
| Lymphocytes, giga/L *(NA= 47)* | 1.9 ± 6.7 | 1.5 ± 1.7 | 1.2 ± 1.1 | 1.8 ± 0.7 | 1.1 ± 0.5 | **0.0003** |
| Eosinophils, giga/L *(NA=48)* | 0.121 ± 0.112 | 0.158 ± 0.149 | 0.164 ± 0.286 | 0.298 ± 0.237 | 0.237 ± 0.287 | **0.007** |
| Hemoglobin, g/dL *(NA=36)* | 11.5 ± 1.53 | 11.6 ± 2.2 | 11.1 ± 2.2 | 13.9 ± 1.8 | 13.7 ± 2.1 | **0.0001** |
| Platelets, giga/L *(NA= 39)* | 236 ± 119 | 264 ± 131 | 212 ± 115 | 266 ± 82 | 238 ± 84 | 0.4 |
| CRP, mg/dL *(NA= 92)* | 78 ± 74 | 63 ± 67 | 97 ± 95 | 31 ± 41 | 11 ± 6 | **0.0002** |
| Flexible bronchoscopy |  |  |  |  |  |  |
| Bacteria | 13 (22) | 9 (18) | 5 (15) | 0 (0) | 1 (4) | 0.03 |
| Mycobacteria *(NA= 4)* | 0 (0) | 0 (0) | 0 (0) | 0 (0) | 0 (0) | / |
| Positive viral PCR *(NA= 18)* | 9 (15) | 6 (12) | 17 (50) | 0 (0) | 0 (0) | **0.0001** |
| Fungi | 40 (67) | 11 (22) | 15 (44) | 5 (20) | 4 (16) | **0.0005** |
| *Pneumocystis* cysts | 16 (27) | 0 (0) | 0 (0) | 0 (0) | 0 (0) | **0.0001** |
| Positive *Pneumocystis* PCR *(NA= 75)* | 55 (100) | 18 (50) | 4 (16) | 2 (15) | 1 (10) | **0.0001** |
| *Pneumocystis* PCR copies *(NA= 164)* |  |  |  |  |  |  |
| Colonization | 9 (18) | 18 (61) | 4 (100) | 2 (100) | 1 (100) | / |
| Intermediate | 15 (30) | 6 (33) | 0 (0) | 0 (0) | 0 (0) | / |
| Infection | 27 (52) | 1 (5) | 0 (0) | 0 (0) | 0 (0) | / |
| BAL cellularity, cells/ml *(NA=122)* | 282,266 ± 468,411 | 272,437 ± 419,789 | 180,166 ± 116,757 | 256,333 ± 2314,27 | 178,857 ± 86,616 | 0.9 |
| Cell population on BAL, % *(NA=2)* |  |  |  |  |  |  |
| Macrophages | 36 ± 17 | 42 ± 18 | 47 ± 16 | 40 ± 20 | 45 ± 17 | 0.04 |
| Lymphocytes | 54 ± 21 | 51 ± 18 | 46 ± 18 | 53 ± 20 | 51 ± 19 | 0.3 |
| Neutrophils | 9 ± 12 | 4 ± 5 | 5 ± 8 | 5 ± 7 | 2 ± 6 | 0.04 |
| Eosinophils | 1.0 ± 2 | 3.0 ± 7 | 1.3 ± 3 | 1.7 ± 2 | 0.7 ± 1 | 0.04 |
| Morphological abnormalities |  |  |  |  |  |  |
| Activated lymphocytes | 58 (98) | 45 (91) | 33 (97) | 25 (100) | 25 (100) | 0.3 |
| Macrophages into cohesive clusters | 59 (100) | 47 (96) | 34 (100) | 25 (100) | 25 (100) | 0.2 |
| Epithelioid transformation of macrophages | 57 (96) | 46 (94) | 33 (97) | 25 (100) | 25 (100) | 0.8 |
| Foamy macrophages | 39 (66) | 39 (80) | 30 (88) | 21 (84) | 20 (80) | 0.1 |

Data are presented as mean ± SD or N (%). ***** Multiple testing issue was tackled using Benjamini-Hochberg method by limiting False Discovery Rate to 5%. Statistical significance threshold was at 3%.

Abbreviations: DILD Drug-induced lung disease; HP Hypersensitivity pneumonitis; N number; NA not applicable; PCP *Pneumocystis* pneumonia
